# Acid-base potentiometric titration using a stainless steel electrode without oxidative treatment

**DOI:** 10.55730/1300-0527.3580

**Published:** 2023-07-19

**Authors:** Javier E. VILASÓ-CADRE, Daniel BENÍTEZ-FERNÁNDEZ, Ilse A. LÓPEZ-ÁLVAREZ, F. Yuliana TOVAR-VÁZQUEZ, María A. ARADA-PÉREZ, Iván A. REYES-DOMÍNGUEZ

**Affiliations:** 1 Institute of Metallurgy, Autonomous University of San Luis Potosi, San Luis Potosi, Mexico; 2 Department of Chemistry, Faculty of Natural and Exact Sciences, University of Oriente, Santiago of Cuba, Cuba; 3 National Council of Science and Technology (CONACYT), Mexico City, Mexico

**Keywords:** Potentiometry, titration, pH, stainless steel, electrode

## Abstract

An AISI 304 stainless steel laminar electrode without oxidative treatment was investigated for the potentiometric titration of hydrochloric acid with sodium hydroxide. The proposed electrode was obtained from metalworking cuttings. Scanning electron microscopy, energy dispersive X-ray spectroscopy, X-ray fluorescence spectroscopy and X-ray diffraction were used to study the surface morphology and chemical composition of the electrode. The electrode showed a sensitivity of 59.18 ± 0.37 mV/pH, which was reproducible under intermediate conditions. Potentiometric titration showed a curve with deviations from pH 9.5 with respect to the glass electrode. However, this did not affect the quantification as the jumps of the curves coincided. The endpoint was 9.25 mL for both electrodes and the hydrochloric acid concentration was 0.0845 mol/L, with a deviation of 0.0004 mol/L from the standard concentration of 0.0841 mol/L. The nonartificially oxidised electrode did not show any crystalline oxide phases, whereas after oxidation it showed semicrystalline phases of iron and chromium oxides and increased the crystallinity of the steel. Despite the low content of surface oxides, stainless steel electrodes can give a Nernstian response to pH, depending on the surface characteristics of the material. This leads to the need to calibrate any electrode prior to oxidative treatment to rule out a Nernstian response without surface modification.

## 1. Introduction

Potentiometry is an electroanalytical method based on the measurement of the potential difference in a galvanic cell consisting of an indicator and a reference electrode. Direct potentiometry allows the quantification of several ions and molecules by sensing with a selective electrode. However, potentiometry can also be used to indicate the endpoint of a titration, known as indirect potentiometry or potentiometric titration [1–3].

Potentiometric titration has advantages over visual indication because it is a more accurate and robust technique with special utility in coloured samples, oily samples and many others where visual indicators cannot be utilised [4–6].

In potentiometric titrations, the analytical response is the measured potential (E) or pA versus the added volume (V), where A is a cation, anion, or molecule, including hydrogen ion (pH). pA is the negative logarithm of the concentration of A, which means that the measured potential should depend on the concentration of A, so the indicator electrode must be sensitive and selective for the analyte to be determined. Although acid-base potentiometric titrations are the most used, all types of volumetric titrations can be carried out using this instrumental indication [7].

The most common pH transducer is the glass electrode, but sensitive response has also been achieved using metal oxide (MO) and metal/metal oxide (MMO) electrodes. RuO_2_, IrO_2_, TiO_2_, ZnO, SnO_2_, PtO_2_, WO_3_, and many others have been reported for potentiometric hydronium sensing [8–12]. These metal electrodes have several advantages over glass: lower cost, miniaturisation is possible, mechanical and chemical stability, low interference from secondary monovalent ions, long term storage at atmospheric conditions, etc. [11].

Modification of the electrochemical double layer by specific adsorption of H^+^ ions has been proposed as the mechanism underlying potentiometric pH sensing using the electrodes discussed above [11]. This opens up the possibility of using some materials whose solid-solution interface could also be modified by the hydronium ion, and research focused on inexpensive materials could reduce the cost of analysis compared to using the glass electrode.

Stainless steel is a group of metallurgical materials based on steel alloys containing at least 10.5% chromium and other elements such as nickel, manganese, silicon, phosphorus, and sulphur [13,14]. The use of stainless steel as a pH electrode has been studied for a long time. However, the material has usually been subjected to oxidative treatment either thermally or chemically. Normura et al. [15] show the use of AISI 304 and AISI 316 stainless steels for the quantitative detection of hydronium ion. The steels were treated by heating between 400 and 700 °C for 1 h or by immersion in 2.5 mol/L CrO_3_ and 5 mol/L H_2_SO_4_ at 70 °C for 10–30 min. Zampronio et al. [16] used a stainless steel electrode for the determination of acid mixtures by flow injection analysis. Before the electrochemical application, the electrode was immersed in 2.5 mol/L K_2_Cr_2_O_7_ and 5 mol/L H_2_SO_4_ at 70 °C during different times.

More recent research has tried to elucidate aspects of the response of stainless steel to pH, as the sensing mechanism is not entirely clear. For example, Hashimoto et al. [17] showed that the layer underlying an oxide coating plays a key role in the response; therefore, the oxide-H^+^ double layer interface is not the only one responsible.

The fact is that despite oxidative treatment, the calibration response is not always Nernstian [16], which suggests that the surface characteristics of the steel play a fundamental role. Despite this, there are few studies on the use of the nonoxidised surface of stainless steel as a pH sensor. Research that has performed the evaluation without oxidation has not found a better potentiometric response than when oxidation is performed [17]. However, this work presents evidence that the nonartificially oxidised surface of an AISI 304 stainless steel electrode can respond in a Nernstian manner to pH. The study focuses on the application of the nonoxidised steel in an acid-base potentiometric titration.

The use of a stainless steel electrode for pH detection is economically viable because residual parts such as cuttings can be used. This is dramatically less expensive than using a glass electrode. This has led to a renewed interest in this type of electrode, especially for experiments that are not suitable for glass electrodes, such as measurements on small sample volumes or conditions where there is a risk of membrane rupture [17].

## 2. Materials and methods

### 2.1. Reagents

Reagent grade sodium hydroxide (≥98%) and hydrochloric acid (37%) were acquired from Sigma Aldrich for the experiments. Deionised water from the Hydronix purification system was utilised (0.055 μS/cm).

### 2.2. Experimental electrode

A 4 cm × 0.5 cm × 0.5 mm (length × width × thickness) laminar piece of stainless steel, scrap from metalworking cuttings, was used as an indicator electrode in the potentiometric experiments. The electrode was washed with deionised water and dried with paper towels before being characterised and used in potentiometric experiments.

### 2.3. Characterisation of the stainless steel electrode

The surface morphology of the electrode was examined using a JEOL JSM-6610LV scanning electron microscope (SEM) at 20 kV. Energy dispersive X-ray spectroscopy (EDS) was used to qualitatively study the chemical composition of the alloy. Elemental quantification was performed by X-ray fluorescence spectroscopy (XRF) using an Olympus Delta Professional portable XRF analyser.

### 2.4. Potentiometric measurement

The electrochemical cell consisted of the stainless steel indicator electrode and a Thermo Scientific Orion Ag/AgCl reference electrode, both immersed in a 50-mL beaker. The stainless steel electrode was calibrated by measuring the potential of buffer solutions, the pH of which had previously been adjusted using a Thermo Scientific Orion glass pH combination electrode. Measurements were made using a Thermo Scientific Orion Star A211 pH meter.

To evaluate the intermediate precision of the potentiometric calibration of the stainless steel electrode, the procedure was repeated on another day, with the preparation of new buffer solutions of different pH. The curve obtained was compared with the previous one using a statistical procedure for comparing linear regression curves.

### 2.5. Potentiometric titration

The potentiometric titration of 0.0841 mol/L HCl with 0.0914 mol/L NaOH was carried out using the stainless steel electrode. The HCl solution was standardised by titration against sodium carbonate, while the NaOH solution was standardised against oxalic acid [18]. For the potentiometric titration, 10 mL of HCl were added to a beaker, the electrodes were placed in the beaker and completely covered with water. The pH was then measured with stirring after each addition of NaOH from a burette. The experimental results were compared with those obtained using the glass electrode and with visual titration using bromothymol blue as an indicator.

### 2.6. Surface characteristics compared to the oxidised electrode

The surface characteristics of stainless steel that allow it to respond to pH without oxidative treatment were studied by X-ray diffraction (DRX) in comparison with the material oxidised at 700 °C for 1 h in a furnace [15]. The diffractograms were obtained with a Bruker D8-Advance instrument operated at 30 kV using a wavelength of 1.5406 Å (CuKα). Diffraction was performed in the narrow beam mode from 2θ values of 10° to 70°. A Canon EOS 500D camera was used to take high-resolution photographs of the electrode surface before and after oxidation.

## 3. Results and discussion

### 3.1. Characterisation of the stainless steel electrode

**Figure 1:**
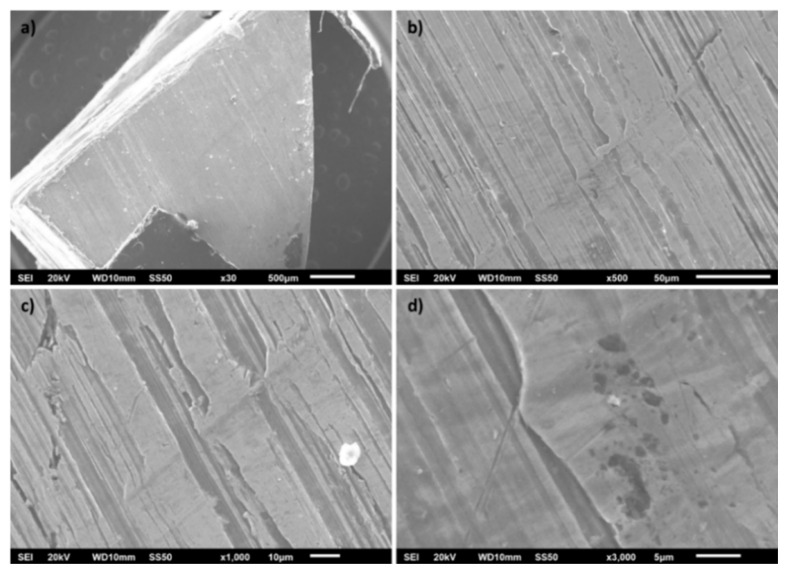
Scanning electron micrographs of the stainless steel electrode used for the potentiometric acid-base titration.

The SEM micrographs of the electrode surface at various magnifications are shown in Figures 1a–1d. Figure 1a gives an overview of the morphology of the entire electrode section. At this magnification, grooves are already visible, which can be seen in greater detail in Figures 1b and 1c, where scratches can also be observed [19]. Such surface micro-irregularities are produced when steel is cut [20]. Figure 1d shows surface defects that are typically caused by gas entrapment during the casting of the material [21,22]. These features are important because rough surfaces have a larger electroactive area than polished surfaces, which can improve the electrochemical response. Chen et al. [23] show how increasing the roughness factor on Pt electrodes results in a larger electroactive surface area. De Levie [24] deals with the influence of the surface roughness of solid electrodes on electrochemical measurements. They demonstrated the deviation of the impedance measured on rough electrodes from the behaviour expected for a flat electrode.

**Figure 2:**
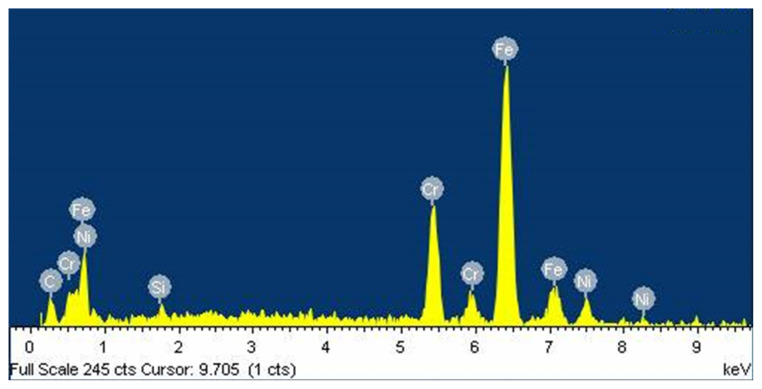
Energy dispersive X-ray spectrum of the stainless steel electrode used for the potentiometric acid-base titration.

Figure 2 shows the EDS spectrum of the electrode, several elements have been identified, including iron, carbon, and chromium, as expected for stainless steel [25].

A quantitative characterisation of the stainless steel can be made using the XRF method. The results of the elemental composition by this method are presented in Table 1. Based on the standards ASTM A240/A240M – 11 [26], ASTM A895 – 89 [27], and the experimental composition reported by Dalipi et al. [28], it can be stated that the steel used in our work could be either AISI 303 or AISI 304, the possibility of both types is due to the fact that their respective compositions are very similar. However, Drake and MacDonald [29] state that the sulphur content is a distinguishing criterion between AISI 303 and 304 steels, and it can be seen in Table 1 that the sulphur content of our electrode is less than 0.04%, which corresponds to the standardised composition reported for an AISI 304 stainless steel, allowing to conclude that the experimental steel matches this type.

### 3.2. Stainless steel electrode calibration for pH measurement

**Figure 3:**
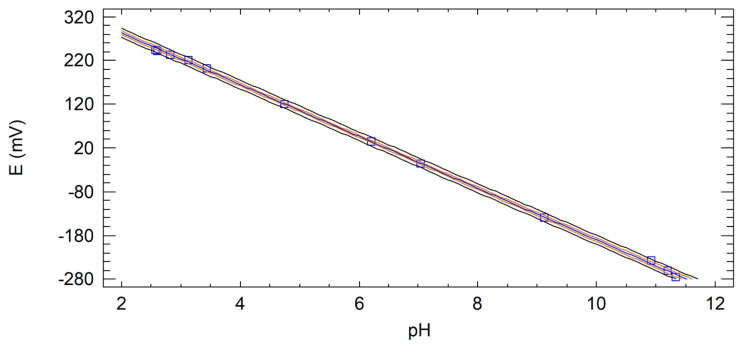
Calibration curve for the pH measurement using the AISI 304 stainless steel electrode.

Figure 3 shows the calibration curve for the pH measurements using the stainless steel electrode without oxidative treatment. A linear fit with the confidence intervals for 95% is shown, the trend indicates that the Nernst equation (Eq. 1) is satisfied for the data series [30].

Stainless steel is normally subjected to an oxidative treatment before being used as a pH electrode. However, several authors have described the formation of a passivating chromium oxide layer on the surface of stainless steel as a corrosion protection mechanism [14,25,31]. This is due to the high ionisation tendency of chromium relative to iron [14]. This oxide layer may be the cause of the response of nonartificially oxidised stainless steel to H^+^ ion via an electrochemical double layer mechanism. According to the theory proposed by several authors, there are binding sites on the surface of oxidised metals that allow the specific adsorption of H^+^, generating a potential difference depending on the concentration of these ions, which, from an analytical point of view, leads to pH sensing [11].


(1) $$E=E^{0^{\prime }}-S\times pH$$

where E is the measured potential (mV); E^0’^ is the intercept of the calibration (mV), representing the formal potential; S is the slope of the calibration, whose ideal value is 59.16 mV/pH at 298 K; and pH is −log[H^+^], where [H^+^] is the concentration of H^+^.

Further conclusions can be drawn by analysing the linear regression statistics shown in Table 2. The absolute value of the calibration slope indicates the sensitivity of the potentiometric transducer. A pH electrode with an absolute calibration slope equal to 59.16 mV/pH has a Nernstian response, i.e. it has an ideal response for a temperature of 298 K. If the calibration slope has an absolute value significantly lower than 59.16 mV/pH, it is said that there is a sub-Nernstian response of the sensor, which is not desirable as it results in lower sensitivity. If the absolute slope is greater than 59.16 mV/pH, there is a super-Nernstian response, i.e. greater than expected according to the Nernst equation. In the case of the stainless steel electrode studied, it is observed that the slope has an absolute value of 59.18 ± 0.37 mV/pH, very close to the ideal, which allows the response to be classified as Nernstian, indicating that the proposed electrode without oxidative treatment is sufficiently sensitive to the hydronium ion.

According to Pearson’s coefficient, there is a strong correlation of 99.98% between the measured potential and the pH, the negative sign indicates that the correlation is indirect, which is clearly seen in Figure 3 where the potential decreases with increasing pH. The coefficient of determination (R^2^) indicates that the mathematical model of the regression explains 99.96% of the variability of the data series. The regression coefficient adjusted for degrees of freedom (R^2^_adj._) is useful for comparison with calibrations performed by other authors.

Analysis of variance (ANOVA) is an important test to draw conclusions about the statistical significance of the calibration (Table 3). In this case, the p-value is significantly lower than the significance level of the test (0.05), indicating that the data series fits a line with statistical significance, which validates the calibration of the AISI 304 stainless steel electrode and therefore the sensitivity expressed as the absolute slope of the curve.

Table 4 shows the hypothesis test for the comparison between the slope obtained in the calibration and the expected ideal value for the monoelectronic transfer according to the Nernst equation. As the p-value is higher than the significance level of the test, it can be stated that there is no statistical difference between the experimental slope and the ideal value. This confirms that the electrode response is Nernstian.

**Figure 4:**
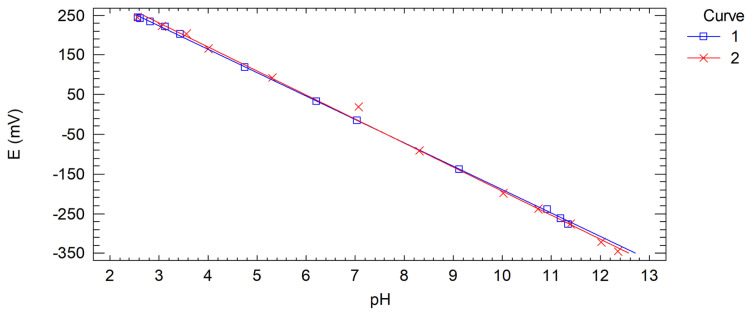
Potentiometric calibration curves of the AISI 304 stainless steel electrode obtained on different days with different pH values of the buffer solutions.

In order to be sure that the electrode shows a Nernstian response to pH whenever it is used, the intermediate precision of the calibration was evaluated, i.e. the procedure was repeated by varying some experimental conditions. Figure 4 shows the calibration curve already discussed, which has been identified as 1, and a new curve obtained on another day with buffer solutions of different pH values, this plot has been identified as 2. It can be seen that both regression curves coincide, which can be confirmed by a comparison test.

Table 5 shows the ANOVA for the comparison of the two regression curves generated in the calibrations. As the p-value for the slopes is greater than 0.1, there is no statistical difference at the 90% confidence level or higher. Similarly, the p-value for the intercepts is greater than 0.1, and thus, there is also no statistical difference between these two regression parameters. Therefore, the Nernstian potentiometric response of the steel electrode is reproducible on different days and using different buffer solutions. This indicates that the observed Nernstian behaviour is inherent to this electrode.

The sensitivity of the AISI 304 stainless steel electrode studied is better than that of other reported nonglass electrodes. Huang et al. [32] present a sensor for pH based on the iridium oxide sensing film; the sensitivity was 51.1 mV/pH. Santos et al. [33] report a conformable pH sensor based on WO_3_ nanoparticles; the sensitivity was 56.7 mV/pH. Manjakkal et al. [34] report a thick film pH sensor based on Ta_2_O_5_ with an absolute calibration slope of 45.92 mV/pH. Guo et al. [35] present different tungsten/tungsten oxide electrodes for long-term pH monitoring, the absolute slopes of the different transducers were sub-Nernstian, with values between 47.2 and 50.2 mV/pH.

### 3.3. Potentiometric titrations

**Figure 5:**
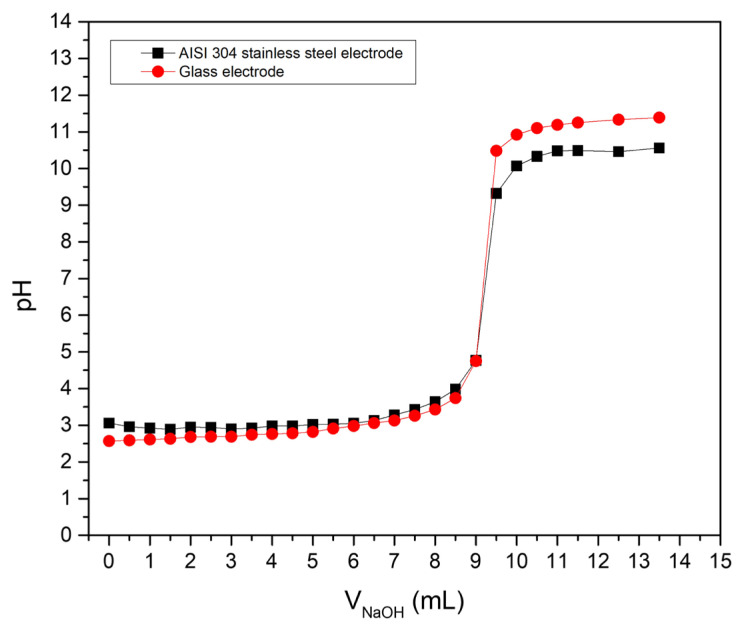
Potentiometric titration curves of HCl with NaOH using the AISI 304 stainless steel electrode and the glass pH electrode.

Figure 5 shows the potentiometric titration curves of HCl with NaOH using the AISI 304 stainless steel electrode and the glass pH electrode. The titrant was added in 0.5 mL portions.

In general, a submeasurement of the pH values with respect to 7 is observed when using the stainless steel electrode, resulting in a more closed curve with respect to that obtained with the glass electrode. However, this is not as pronounced in acidic and neutral media, contrary to what occurs from pH 9.5 onwards, where a more important difference is observed. This behaviour has been commonly described for MO and M/MO electrodes; in fact, some authors mention the deviations in basic media as one of the disadvantages of this type of electrodes [11]. Nevertheless, in a potentiometric titration the objective is not the direct measurement of pH; therefore, this is not a problem if there is correspondence in the jump that occurs at the equivalence point, something that can be seen more clearly in the first and second-derivative curves.

**Figure 6:**
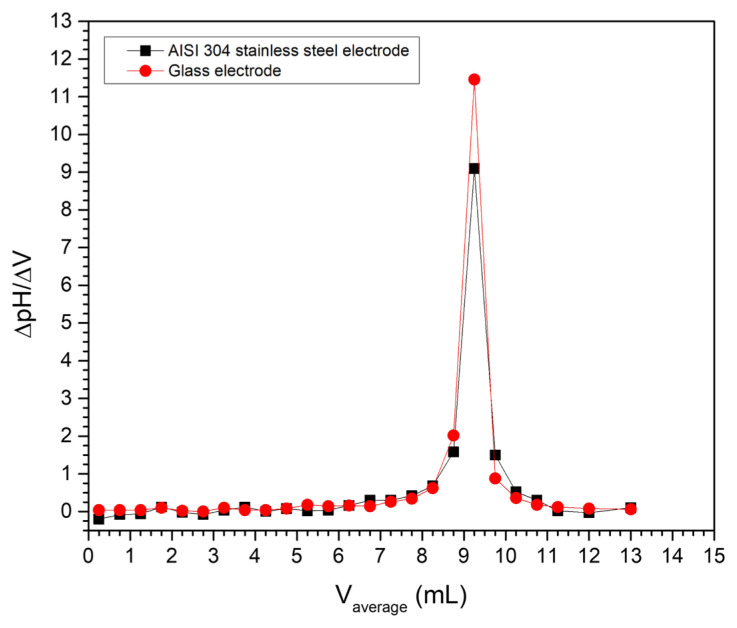
First-derivative method for the potentiometric titration of HCl with NaOH using the AISI 304 stainless steel electrode and the glass pH electrode.

**Figure 7:**
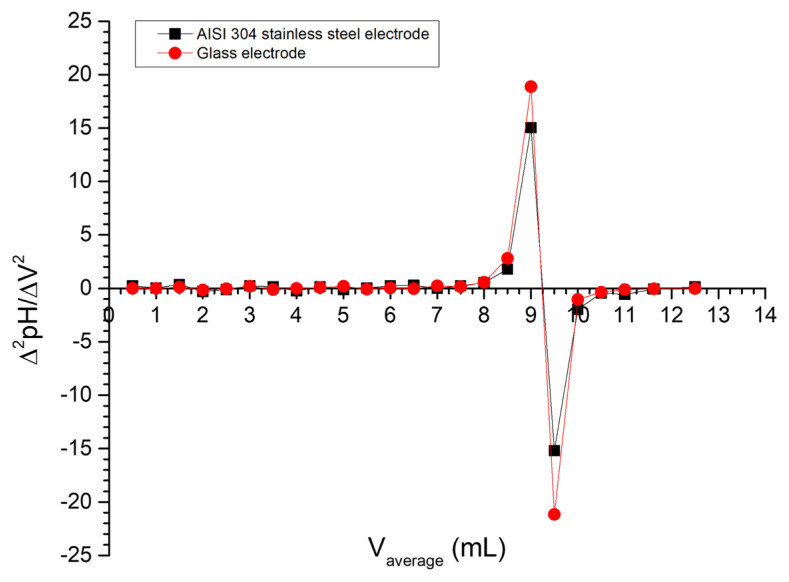
Second-derivative method for the potentiometric titration of HCl with NaOH using the AISI 304 stainless steel electrode and the glass pH electrode.

Figure 6 shows the first-derivative method graph for the potentiometric titrations using the stainless steel electrode and the glass electrode. The first-derivative plot of a titrimetric curve produces a data series along the abscissa axis showing a well-defined peak corresponding to the endpoint [36]. In our data series there is a high agreement between the peaks, indicating the correspondence of the endpoints using both electrodes. A second derivation of the potentiometric curve allows the endpoint to be seen more accurately as an intercept with the abscissa axis [36]. Figure 7 shows this method for the acid-base titration using the stainless steel electrode and the glass electrode. The volume at the endpoint for both curves is 9.25 mL, which corresponds to the result of the first-derivative method.

Table 6 shows the results of the acid-base titrations using visual and potentiometric indication. As described above, the volume at the endpoint, and therefore the concentration, is the same for both electrodes. The concentration obtained with the instrumental indication shows a deviation of 0.0004 mol/L with respect to the standardised concentration, while for the visual method it is 0.0009 mol/L, demonstrating a smaller deviation in the potentiometric titration. The ideal indication method for a titration should give an endpoint volume equal to the equivalence volume, but in practice this is impossible because all methods have systematic errors that lead to some difference between the endpoint and the equivalence point of the reaction, this difference is known as the titration error [37–40]. However, some methods are more accurate than others, and potentiometric indication is much more accurate than visual indication because the latter is subject to more errors from both the method and the analyst. Among the most important method errors are those related to the colour change of the indicator. Often the equivalence pH of the strong acid-strong base neutralisation reaction is not within the colour change range of the indicator used, resulting in a small difference between the added volume from the burette and the equivalence volume. This type of error can be minimised by selecting the best indicator, e.g., bromothymol blue, or by using a blank correction. On the other hand, there are analyst errors. The most difficult to correct is related to the ability of the analyst to detect the colour change of the indicator. This error depends on the visual skill to distinguish a colour change, which usually introduces uncertainties of ± 0.5 to ± 0.1 pH units. Another important analyst error is that caused by incorrect reading of the volume in the burette due to the position of the eyes in relation to the scale, known as the parallax error, which can be minimised with good practice [37].

### 3.4. Surface characteristics compared to the oxidised electrode

To draw further conclusions on the use of the AISI 304 stainless steel electrode without oxidative treatment, the composition of the crystalline phases on the surface was studied in comparison with the same electrode after being subjected to an oxidative treatment at 700 °C for 1 h. This makes it possible to describe the unmodified surface used in the potentiometric experiments with respect to the oxide coating commonly used on stainless steel electrodes.

Recent research has revived the use of stainless steel electrodes as an inexpensive and durable option for pH measurement, especially in applications where the glass electrode must be discarded [17]. However, the fact that not everything is clear about the pH response of these electrodes leaves some aspects to be investigated.

**Figure 8:**
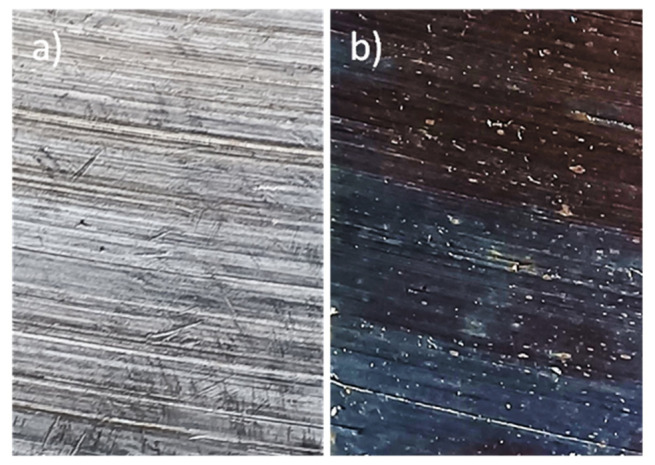
a) AISI 304 stainless steel surface before the oxidative treatment at 700 °C for 1 h, b) AISI 304 stainless steel surface after the oxidative treatment.

Common oxidation methods for stainless steel electrodes are heat treatment between 500 and 900 °C or chemical attack with a mixture of CrO_3_ and H_2_SO_4_ at 70 °C [15–17]. Some authors have shown that the best heat treatment is at 600 to 700 °C [15,17]. Figure 8 shows the high-resolution photographs of the steel surface before and after the oxidation treatment at 700 °C for 1 h. There are marked differences in the colours, indicating that the surface has indeed been covered with an abundant layer of oxides. The brown and blue-green colours of the thermally oxidised surface match those described by other authors, indicating that the surface composition is not homogeneous after oxidation [17].

**Figure 9:**
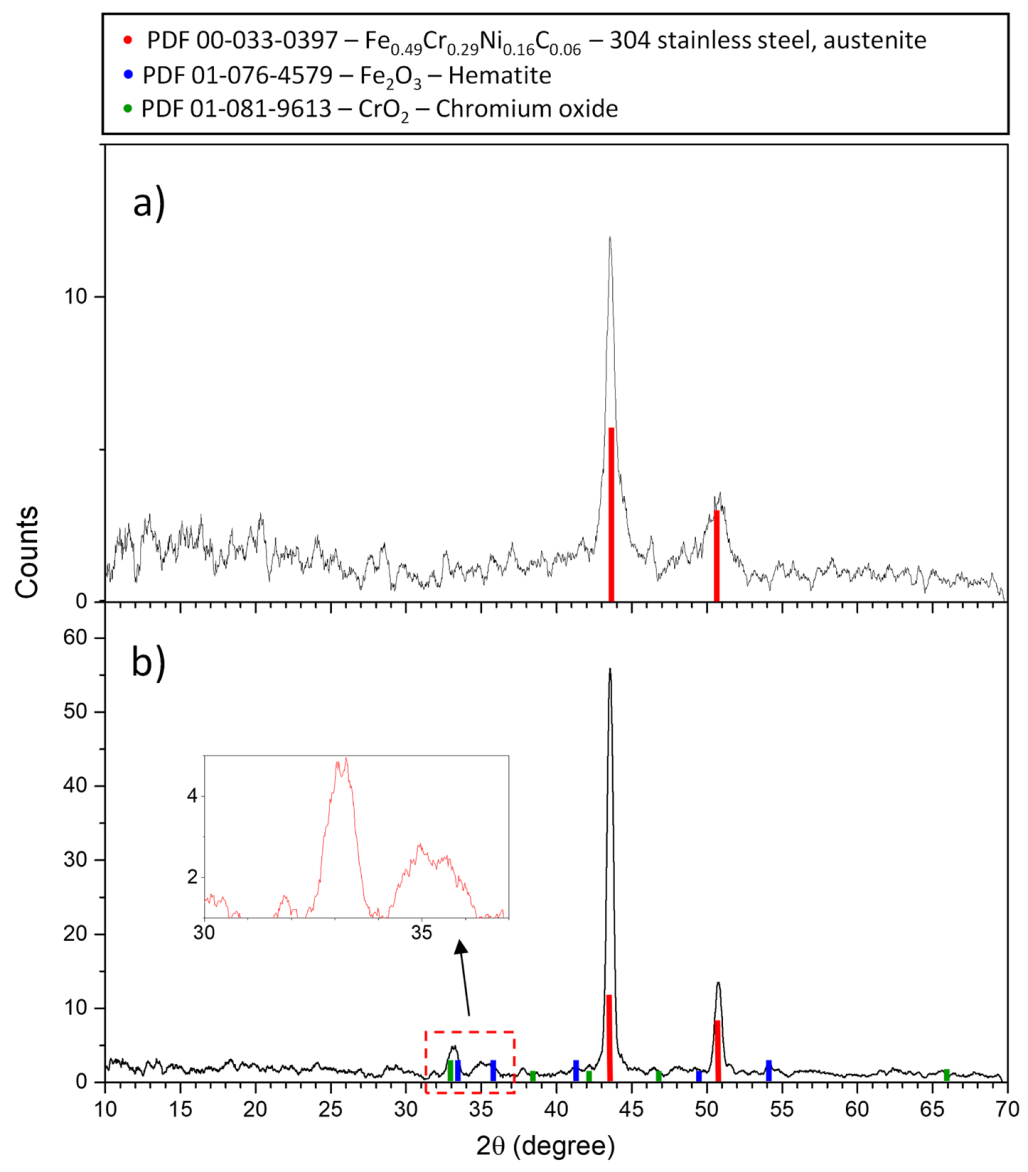
XRD pattern of the stainless steel electrode: a) before oxidative treatment, b) after oxidation at 700 Â°C for 1 h.

Figure 9a shows that the nonheat-treated stainless steel surface has slightly amorphous characteristics due to the width of the peak at 51° and the slight broadening of the peak at about 44°, both corresponding to the austenitic phase of the steel which has a face centred cubic (fcc) structure [17]. These peaks become sharper and more intense with treatment, indicating an improvement in the crystallinity of the steel. No oxide crystalline phases are detected in the steel without oxidative treatment, possibly for two reasons: firstly, the passivation layer is very thin in nonoxidised steel; therefore, the amount present may be below the detection limit of the XRD method; and secondly, the content may be amorphous, which, in conjunction with the former, limits the observation of any small bands. The absence of a signal for any crystalline or amorphous surface oxide phase in the nontreated steel is in agreement with the result obtained by other authors [17, 41]. However, it has been widely described that the corrosion of stainless steel is prevented by the thin oxide layer on the surface. In a study by Hashimoto et al. [17] on the effect of oxidation of AISI 304 stainless steel on the potentiometric response to pH, they found that the Nernstian response was achieved with heat treatment at 600 °C for 24 h, while the nontreated steel failed to reach the ideal sensitivity (59.16 mV/pH), contrary to our results where a Nernstian calibration slope of 59.18 ± 0.37 mV/pH was obtained. These authors also found that although the electrode is coated with an oxide layer after heat treatment, the composition of the underlying layer plays the major role in the pH response. This gives some idea of why stainless steel did not show a Nernstian sensitivity for these authors, whereas it did in the present work. If the natural passivation layer has different characteristics between two pieces of AISI 304 stainless steel, then the pH response may also be different.

Figure 9b shows the appearance of the crystalline phases of chromium (IV) oxide and hematite after heat treatment, demonstrating that the surface has indeed undergone chemical modification; it also provides compositional details of this modification. The presence of other oxides, such as Cr_2_O_3_ and Fe-Cr mixed phases, has been reported in stainless steels after treatment, which did not occur in our material [17,41]. The diffractogram section within the red dashed box in Figure 9b has been enlarged to allow a closer look at the peaks located approximately at 33° and 35°, which show some broadening, indicating that the oxides formed are semicrystalline due to the fact that the heat treatment time was not sufficient to produce fully crystalline phases. However, short oxidation times are commonly used because prolonging the heat treatment causes the transformation of the austenitic phase of the steel into martensite, which affects the sensitivity of the material to pH [17].

Figures 9a and 9b have shown that surface oxidation of the steel introduces important differences in composition and crystallinity. This helps to understand that the response of stainless steels to hydronium ions is not entirely related to the oxide layer formed when the steel is artificially oxidised, as this would be expected to be the necessary condition for a Nernstian response and is not the case, the natural thin oxide layer of the steel can also respond in this way, suggesting that this layer may play a fundamental role despite the oxidation. Moreover, some authors even when performing oxidative treatment of steel, achieve sub-Nernstian responses [16]. All this allows us to state that the potentiometric results obtained when a stainless steel electrode is oxidised cannot be generalised, i.e. the presence of an artificial oxide layer does not guarantee a Nernstian response, nor does the absence of oxidation prevent a response of this type. Therefore, each steel electrode must be evaluated by calibration prior to oxidation, which is not done in the reported works, as the oxidation process is almost always carried out and it is assumed that this is sufficient to obtain an adequate potentiometric response.

## 4. Conclusion

Oxidative treatment of the AISI 304 stainless steel electrodes will produce a more extensive and crystalline surface oxide layer, but the Nernstian response can be achieved without treatment depending on the surface characteristics of the material. This leads to the need to calibrate the electrode prior to oxidative treatment to rule out a Nernstian response without surface modification.

AISI 304 stainless steel electrodes are an inexpensive and durable option for monitoring pH in potentiometric titrations, and even more cost-effective if oxidative surface treatment can be omitted. The titrimetric results are comparable to those obtained with potentiometric indication using a glass electrode or with visual indication.

## Figures and Tables

**Table 1:** Elemental composition by X-ray fluorescence spectroscopy of the stainless steel electrode used for the potentiometric acid-base titration. The standardised composition for AISI 303 and AISI 304 stainless steels is also shown.

	Experimental composition		Standardised composition (%)	
Element	Weight (%)	+/−	AISI 303^a^	AISI 304^b^
Fe	70.29	0.21	NR	NR
Cr	17.22	0.15	17–19	17.5–19.5
Ni	9.33	0.15	8–10	8–10.5
Mn	1.75	0.08	≤ 2	≤ 2
Mo	0.48	0.01	-	-
Cu	0.48	0.04	-	-
Si	0.38	0.06	≤ 1	≤ 0.75
V	0.06	0.02	NR	NR
S	< 0.04	-	≥ 0.15	≤ 0.03

a: ASTM A895 – 89,

b: ASTM A240/A240M – 11,

NR: nonreported.

**Table 2:** Statistics of the linear regression for the calibration curve for the pH measurement using the AISI 304 stainless steel electrode.

Statistic	Value
Slope (mV/pH)	−59.18 ± 0.37
Intercept (mV)	401.58 ± 2.62
r	−0.9998
R^2^	0.9996
R^2^_adj._	0.9996

r: Pearson’s correlation coefficient, R^2^: determination coefficient, R^2^_adj._: determination coefficient adjusted for degrees of freedom.

**Table 3:** Analysis of variance for the calibration curve for the pH measurement using the AISI 304 stainless steel electrode.

Source	Sum of squares	DF	Mean square	F-ratio	p-value
Model	488105.0	1	488105.0	25916.98	0.0000
Residue	188.33	10	18.83		
Total	488293.33	11			

DF: degrees of freedom, significance level of the ANOVA: α = 0.05.

**Table 4:** Hypothesis test to compare the experimental calibration slope with the ideal value according to the Nernst equation.

**Null hypothesis**	S = 59.16
**Alternative hypothesis**	S ≠ 59.16
**Calculated t-statistic**	0.1872
**p-value**	0.8549
**Significance level (** **α** **)**	0.05

S: slope of the calibration curve.

**Table 5:** Analysis of variance to compare the calibration curves obtained with the AISI 304 stainless steel electrode on different days with different pH values of the buffer solutions.

Source	Sum of squares	DF	Mean square	F-ratio	p-value
pH	1.08799·10^6^	1	1.08799·10^6^	11999.02	0.0000
Intercepts	10.1652	1	10.1652	0.11	0.7412
Slopes	192.242	1	192.242	2.12	0.1609
Model	1.08819·10^6^	3			

DF: degrees of freedom, significance level of the ANOVA: α = 0.1.

**Table 6:** Results of the titration of HCl with NaOH using visual indication and potentiometric indication with the AISI 304 stainless steel electrode and the glass pH electrode.

Indication	Titration endpoint (mL)	HCl concentration (mol/L)
Bromothymol blue	9.30	0.0850
AISI 304 stainless-steel	9.25	0.0845
Glass	9.25	0.0845

Standardised HCl concentration = 0.0841 mol/L, V_equivalence_ = 9.20 mL.
